# Robotics-Driven Manufacturing of Cartilaginous Microtissues for Skeletal Tissue Engineering Applications

**DOI:** 10.1093/stcltm/szad091

**Published:** 2024-01-13

**Authors:** Isaak Decoene, Gabriele Nasello, Rodrigo Furtado Madeiro de Costa, Gabriella Nilsson Hall, Angela Pastore, Inge Van Hoven, Samuel Ribeiro Viseu, Catherine Verfaillie, Liesbet Geris, Frank P Luyten, Ioannis Papantoniou

**Affiliations:** Prometheus Division of Skeletal Tissue Engineering, KU Leuven, Leuven, Belgium; Skeletal Biology and Engineering Research Center, Department of Development and Regeneration, KU Leuven, Leuven; Prometheus Division of Skeletal Tissue Engineering, KU Leuven, Leuven, Belgium; Biomechanics Research Unit, GIGA In Silico Medicine, GIGA institute, University ofLiège, Liège, Belgium; Department of Development and Regeneration, Stem Cell Biology and Embryology, KU Leuven, Leuven, Belgium; Prometheus Division of Skeletal Tissue Engineering, KU Leuven, Leuven, Belgium; Skeletal Biology and Engineering Research Center, Department of Development and Regeneration, KU Leuven, Leuven; Prometheus Division of Skeletal Tissue Engineering, KU Leuven, Leuven, Belgium; Skeletal Biology and Engineering Research Center, Department of Development and Regeneration, KU Leuven, Leuven; Prometheus Division of Skeletal Tissue Engineering, KU Leuven, Leuven, Belgium; Skeletal Biology and Engineering Research Center, Department of Development and Regeneration, KU Leuven, Leuven; Prometheus Division of Skeletal Tissue Engineering, KU Leuven, Leuven, Belgium; Skeletal Biology and Engineering Research Center, Department of Development and Regeneration, KU Leuven, Leuven; Department of Development and Regeneration, Stem Cell Biology and Embryology, KU Leuven, Leuven, Belgium; Prometheus Division of Skeletal Tissue Engineering, KU Leuven, Leuven, Belgium; Skeletal Biology and Engineering Research Center, Department of Development and Regeneration, KU Leuven, Leuven; Biomechanics Research Unit, GIGA In Silico Medicine, GIGA institute, University ofLiège, Liège, Belgium; Prometheus Division of Skeletal Tissue Engineering, KU Leuven, Leuven, Belgium; Skeletal Biology and Engineering Research Center, Department of Development and Regeneration, KU Leuven, Leuven; Prometheus Division of Skeletal Tissue Engineering, KU Leuven, Leuven, Belgium; Skeletal Biology and Engineering Research Center, Department of Development and Regeneration, KU Leuven, Leuven; Institute for Chemical Engineering Sciences, Foundationfor Research and Technology–Hellas, Patras, Greece

**Keywords:** animal models, autologous, bone, chondrogenesis, clinical translation, progenitor cells, tissue engineering, Manufacturing, Automation, Image processing

## Abstract

Automated technologies are attractive for enhancing the robust manufacturing of tissue-engineered products for clinical translation. In this work, we present an automation strategy using a robotics platform for media changes, and imaging of cartilaginous microtissues cultured in static microwell platforms. We use an automated image analysis pipeline to extract microtissue displacements and morphological features as noninvasive quality attributes. As a result, empty microwells were identified with a 96% accuracy, and dice coefficient of 0.84 for segmentation. Design of experiment are used for the optimization of liquid handling parameters to minimize empty microwells during long-term differentiation protocols. We found no significant effect of aspiration or dispension speeds at and beyond manual speed. Instead, repeated media changes and time in culture were the driving force or microtissue displacements. As the ovine model is the preclinical model of choice for large skeletal defects, we used ovine periosteum-derived cells to form cartilage-intermediate microtissues. Increased expression of COL2A1 confirms chondrogenic differentiation and RUNX2 shows no osteogenic specification. Histological analysis shows an increased secretion of cartilaginous extracellular matrix and glycosaminoglycans in larger microtissues. Furthermore, microtissue-based implants are capable of forming mineralized tissues and bone after 4 weeks of ectopic implantation in nude mice. We demonstrate the development of an integrated bioprocess for culturing and manipulation of cartilaginous microtissues and anticipate the progressive substitution of manual operations with automated solutions for the manufacturing of microtissue-based living implants.

## Graphical Abstract

**Graphical Abstract:**
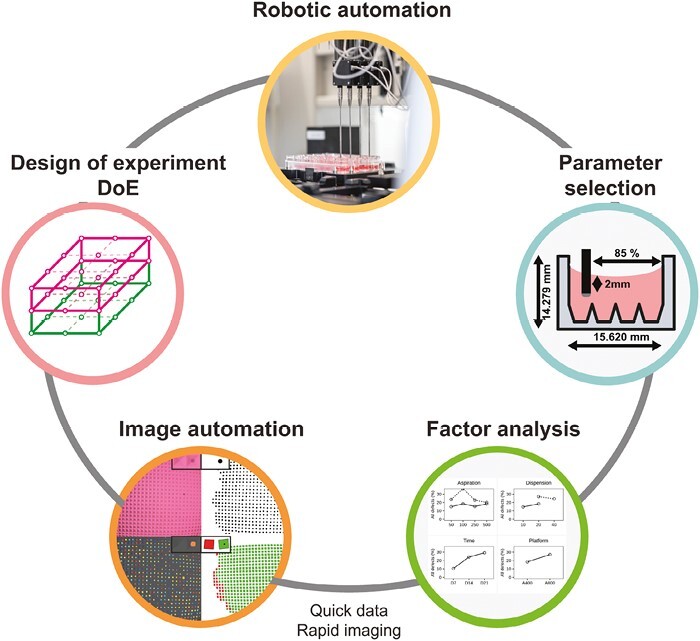

**Significance Statement**

A major bottleneck in skeletal tissue engineering towards preclinical testing, is providing robust automated platforms able to manufacture large quantities of tissue modules, while ensuring high quality microtissue populations. In the present study, we developed a robotic based process for the production of “cartilage intermediate” microtissue populations. Combined with the development of a fully automated image-based detection and segmentation of microtissues in the microwell platform. This work paves the way for the mitigation of unmet clinical challenges through the adoption of robotics -based manufacturing for organoid/microtissue based skeletal implants and living materials.

## Introduction

Spheroids are small 3D structures built by spontaneous aggregation of single cells and they are increasingly used both for drug screening applications^1^ and in the field of regenerative medicine.^2^ As the cells produce their own extracellular matrix and microenvironment, the spheroids can be considered microtissues.^3^ When complex organization and function appear, the term organoid can be used.^4,5^ Compared to 2D monolayer cultures, 3D cultures allow cell growth, matrix deposition, and matrix organization in all directions, which is a more natural environment for cells. In tissue engineering applications, they are ideal as building blocks to create large and complex tissues from the bottom up.^6^ Because of their small size, there are no diffusion limitations on nutrients or growth factors, allowing a more precise control of differentiation.^7^ The current bottleneck in this field is the creation of tissues with sufficient volume and a robust quality profile. To further harness the properties of these cellular building blocks, scalable manufacturing and production in a controlled manner are required to ensure a predefined quality profile.^8^

Microtissue production is achieved by means of different methods including the hanging drop method, drop-seeding cells on low adherence substrates, spinning bioreactors, microfluidics, magnetic aggregation, or the use of nonadherent microwells as reviewed in Liu et al.^4^ Dynamic cultures are easily scalable but harsh on the cells and microtissue size control is difficult.^9,10^ The use of biomaterials both for the creation of spheroids and for downstream steps allows versatile and controlled tissue production, differentiation, and growth.^11,12^ Hydrogels can be produced in-house with tunable shapes, sizes, mechanical, and biological properties, making it an attractive option for research purposes.^13^ Due to a limited number of relevant clinical-grade biomaterials, the use of sacrificial hydrogels^14^ or scaffold-free approaches is also promising. However, these platforms lack scalability and are prone to production errors.

On the other hand, many commercial microwell systems are available, including EZSPHERE, AggreWell, Elplasia plate, SpheroFilm, SphericalPlate 5D, and 3D Petri Dish. Currently, microtissue production in microwell platforms requires manual handling and pipetting, which is prone to errors, including microtissue escape from microwells, followed by uncontrolled agglomeration and thus, batch failure. Monitoring of microtissue cultures and their morphometric quality profiles can be done noninvasively through imaging. Software is already available to segment and analyze microtissues in hydrogel microwells and floating cultures, even enabling the selection of desirable microtissues and studying their fusion kinetics.^15-19^ Commercial microwell systems are typically produced from polycaprolactone (PCL) or polydimethylsiloxane (PDMS) in different microwell shapes, which interact with light, creating complex backgrounds in brightfield images. Manual segmentation using, for example, fiji^20^ or napari^21^ is time-consuming and person dependent, but well suited for small datasets and varying input images.^22-25^ Automated segmentation is fast, reproducible, and can be done with minimal supervision. Yet, for complex images, we still need methods for automated segmentation to monitor microtissue cultures.

Recently, the use of chondrogenic microtissues and organoid assemblies has shown promising results in long-bone defect regeneration through endochondral ossification following the paradigm of developmental engineering.^26-28^ Periosteum-derived cell aggregates form transient cartilage microtissues in chondrogenic medium (CM) containing BMP2, BMP6, GDF5, bFGF2, and TGFβ1.^29^ Bone-forming potency was shown ectopically in nude mice for both individual spheroids as larger constructs and a proof-of-concept was provided for the successful healing of a murine critical-sized long bone defect.^30^ The critical next step is bridging the gap toward large animal models in a preclinical phase. For long bone defects, ovine models are well suited regarding biological similarity, long bone dimensions, and mechanical loading during normal behavior.^31^ A transition to preclinical studies and industrial translation requires a significant scale-up of this bottom-up strategy beyond what is possible by manual methods.

A well-characterized process and automated manufacturing line are imperative toward successful translation.^32-36^ In recent years, significant progress has been made in automating several aspects of tissue-engineered advanced therapy medicinal product (TE-ATMP) manufacturing. Benchtop pipetting robots are used to standardize sample preparation^37,38^ and microwell platform production.^39^ Multidevice platforms have been developed for high content screening of 2D expansion^40-42^ and differentiation,^43-46^ thus showing the feasibility and advantages of automating manual processes. For more complex 3D tissues, robotic culture either generates highly variable organoids^45^ or a low quantity of controllable-size organoids.^12,47^ Furthermore, robotic platforms using microtissues to create complex tissue constructs are appearing.^48-51^ Yet, robotic production of microtissues, with noninvasive methods for monitoring, for these applications is hardly investigated. Apart from their intended use, academic high-content screening facilities can also be explored as manufacturing facilities as a bridge between lab-scale manual microtissue production and large-quantity preclinical scale.

In this study, we tested our high-content screening facility as a platform to automate microtissue production. Specifically, we examined the integration of robotics to automate media changes and noninvasive brightfield imaging for cartilaginous microtissue differentiation in parallel with automated analytics essential for robust manufacturing of TE ATMPs.^52-54^ Moreover, Design of experiment (DOE), goal-oriented statistical approaches for defining factor importance toward predefined critical quality attributes, have been used successfully in ATMP research.^55,56^ DOE approaches are used for optimizing differentiation protocols,^29,57-59^ 3D scaffolds design,^60-62^ and scale-up of cell expansion.^63-65^ In this work, we extend the DOE approach to scalable microtissue production.^29,66,67^ We combine a full factorial DOE approach with noninvasive automated image segmentation and analytics to optimize robotic media changes of cartilaginous bone-forming spheroids in static microwell culture platforms.

## Materials and Methods

### Cell Expansion

Periosteum biopsies were obtained from sheep tibia. After digestion, the cells were cultivated for 8 passages in an expansion medium containing DMEM (Gibco) supplemented with 1% antibiotic-antimycotic (Invitrogen) and 10% FBS (South Afrika FBS, BioWest, France).

### Microtissue Formation

The commercially available microwell platform (AggreWell 800 or AggreWell 400, STEMCELL Technologies Inc., Canada) was coated with Anti-Adherence Rinsing Solution (STEMCELL Technologies Inc.) to avoid cell attachment, centrifuged to ensure homogeneous coating, and washed with basal medium prior to cell seeding. Sheep periosteum-derived cells (sPDCs) were harvested with TrypLE Express (Life Technologies, UK) and seeded at 300 000 cells per 2 mL CM. As the platforms differ in the size of their microwells (1200 microwells, size 400 µm vs 300 microwells, size 800 µm), the resulting microtissues are formed through self-assembly of 250 or 1000 cells for aggrewell400 and aggrewell800, respectively. The cells self-aggregate and were differentiated for 21 days in a serum-free CM containing low glucose DMEM (Gibco) supplemented with 1% antibiotic-antimycotic (Invitrogen), 1 × 10^−3^ M ascorbate-2 phosphate, 1 × 10^−7^ M dexamethasone, 40 µg mL^−1^l-proline, 20 × 10^−7^ M of Rho-kinase inhibitor Y27632 (Axon Medchem), ITS + Premix Universal Culture Supplement (containing 6.25 µg mL^−1^ insulin, 6.25 µg mL^−1^ transferrin and 6.25 ng mL^−1^ selenious acid, 1.25 µg mL^−1^ bovine serum albumin, and 5.35 µg mL^−1^ linoleic acid; Corning), 100 ng mL^−1^ BMP2 (INDUCTOS), 100 ng mL^−1^ GDF5 (PeproTech), 10 ng mL^−1^ TGF-β1 (PeproTech), 1 ng mL^−1^ BMP-6 (PeproTech), and 0.2 ng mL^−1^ basic FGF-2 (R&D systems). Half of the medium was replaced with fresh medium on day 3, 7, 10, 14, and 17.

### Robotic Handling, Automated Medium Changes and Imaging

The Stem Cell Laboratory Automation (STELLA) platform, funded by the NextGenQBio Hercules Foundation grant, was used to create and perform automated protocols for medium change and imaging. It is equipped with 2 liquid handlers (Biomek NX MC and Biomek NXp—Span8; Beckman Coulter), one SCARA robotic arm, one Cytomat 10C Automated Incubator and one Cytomat Microplate Hotel (Beckman Coulter), one High-content Imaging System Image eXpress (Molecular Devices), 2 Plate Delliders (Beckman Coulter), one CapitAll IS Automated Capper/Decapper (Thermo Fisher), one Asymptote Freezer (Grant), and one Sigma 6K15 Centrifuge (Sigma), all inside a BSL2 sterile enclosure. This system enables our group to perform fully automated plate handling, medium change, and imaging based on a design of experiment (DOE) to explore the best conditions for automated pipetting in which the microaggregates are not displaced or aspirated. Briefly, Aggrewell plates containing the microaggregates were manually placed in the incubator 24 hours prior to the automated sequence starts. Plates were then manually moved from the incubator to the Span8 liquid handler where different liquid aspiration and dispensing, needle height, and position were tested according to the DOE. In between the first and second DOE, needle placement was optimized using gelatin microcarriers (CultiSpher S, Percell), which have similar characteristics as living microtissues.

### Automated Image Analysis

Brightfield images of microtissues cultured in Aggrewell were taken manually with an inverted DMi1 microscope (2.5×, 0.07 NA lens, Leica). A custom-made image processing workflow was implemented to automatically segment microtissues and extract quantitative morphological information.

Microtissue segmentation was performed by the pixel classification algorithm of ilastik^66^ software (v. 1.3.3). Brightfield images were annotated with 2 classes (microtissues and background). One image per timepoint per condition was annotated to train the default ilastik classifier (Random Forest with 100 trees and all image filters selected). The training dataset for pixel classification consisted of 4 images where a total of 80 microtissues were manually labeled according to a specific pattern. Images were divided into 4 quadrants and the pixels were assigned to the 2 classes for 6 wells of each quadrant.

The ilastik classifier generated probability maps of each class that were converted to .tif files and transformed into individual objects in cellProfiler^68^ software (v 4.2.1). First, individual (primary) objects were identified by global Otsu thresholding of the microtissue class and declumping to distinguish touching objects. Later, the identified objects were filtered by diameter, compactness, eccentricity, and area to remove small or irregular objects.^68^ Finally, several morphological features of the filtered objects (ie, area and shape) were measured.

Besides segmenting microtissues, another image processing workflow was run in parallel to detect the microwells and localize microtissues in the culture plate. The ilastik classifier was also trained to segment the microwell boundaries from the background. The probability maps generated by ilastik were analyzed with the Python library OpenCV^69^ detect the boundary points of each microwell. Then, the sp package in R^70^ was used to match microtissue locations to microwell locations. Microwells were considered empty if the centroid of no microtissue laid within the boundary points.

### Particle Distribution Analysis

Following microtissue morphological measurements, separate datasets were created for each platform × timepoint combination, thus including microtissues identified in a 24-well plate. Spherical shape was assumed to calculate a predicted volume per microtissue (mm³) from the projected area measurement. The microtissues were ordered based on their size, followed by the calculation of the cumulative volume and the identification of particle distribution parameters. D90 represents the individual microtissue volume where 90% will be smaller. Similarly, D50 represents the volume where 50% is larger and 50% is smaller, while D10 represents the smallest portion where 10% is smaller. The span is a particle distribution measurement of variability for non-normal distributions with potential outliers, calculated as span=(D90−D10)/D50. Finally, the predicted tissue volume, as a yield measurement, was calculated as the number of theoretical microwells per well times the average volume per microtissue, corrected for the number of empty microwells.

### Formation of Macro-Construct

Custom round-bottom macrowells were created in 3% agarose (w/v; Invitrogen) and sterilized using UV. Macroconstructs were created by collecting microtissues from 3 wells. Microtissues were gently flushed out from their microwells on day 21, concentrated, and added to the macrowells to sediment for 1 hour. Aggrewell400 implants consist of ~3600 microtissues, while the aggrewell800 implants consist of ~900 microtissues, yet both are the result of 9 × 10^5^ cells at day 0. CM, as previously described, was added, and the constructs were incubated for 24 hours at 37 °C, 5% CO2, and 95% humidity to fuse into a coherent implant.

### In Vivo Ectopic Implantation

In vivo bone forming potency assessment was performed through ectopic implantations in female immune-compromised mice (Rj:NMRInu/nu, age 6-20 weeks). An incision was made on the back of the mice under general anesthesia (ketamine/xylazine) by intraperitoneal injection to create 2 pockets at the shoulder region per mouse. Implants were removed from the agarose macrowell, and washed in 1xDPBS. Four constructs per condition were implanted subcutaneously in the back at the shoulder region of 4 different female immune-compromised mice (Rj:NMRInu/nu), with one implant per condition per mouse. The incision was closed by staples followed by postoperative administration of buprenorphine as pain relief. After 4 weeks, the mice were sacrificed by cervical dislocation. Then, the implants were taken out and fixed for 4 hours in 4% paraformaldehyde (PFA).

### Micro-CT

3D quantification of mineralized tissue in PFA-fixed explants was done through micro-CT (Pheonix Nanotom M, GE Measurement, and Control Solutions). Explants were scanned with a diamond target, mode 0, 500 ms exposure time, 1 frame average, 0 image skip, 2400 images, and a 0.1-mm aluminium filter. Samples were scanned with a voxel size of 3 µm. CTAn (Bruker micro-CT, BE) was used for all image processing and quantification of mineralized tissue based on automatic Otsu segmentation, 3D space closing, and despeckle algorithm. The percentage of mineralized tissue was calculated with respect to the total explant volume. CTvox (Bruker micro-CT, BE) was used to create 3D visualization.

### DNA Quantification, RNA Isolation and Gene Expression Analysis

DNA content was quantified from cell lysate using the DNA assay kit QuantiT dsDNA HS kit (Invitrogen) according to the manufacturer’s protocol. RNA was isolated from the lysate with the RNeasy Mini Kit (Qiagen) according to the manufacturer’s protocol and quantified with NanoDrop 2000 (Thermo Fisher Scientific). RevertAid H Minus First Strand cDNA Synthesis Kit (Thermo Fisher Scientific) was used for reverse transcription. One microgram oligo(dT18) was added to 11 µL RNA for 5 minutes at 65 °C, the reaction mixture (4 µL 5× reaction buffer, 1 µL ribolock ribonuclease inhibitor, 2 µL dNTPmix (10 × 10^−3^ m), and 1 µL RevertAid H Minus M-MuL VRT) was added, cDNA was generated using the Applied Biosystems Veriti 96-Well Fast Thermal Cycler (60 minutes at 42 °C followed by 10 minutes at 70 °C) and diluted in RNase-free water to 5 ng mL^−1^. Fast SYBR Green Master Mix (Thermo Fisher Scientific), 5 ng mL^−1^ cDNA, and specifically designed primers were used to perform Quantitative reverse transcription polymerase chain reaction consisting of a denaturation step at 95 °C, followed by 40 cycles of 95 °C, 3 seconds and 60 °C, 20 seconds. For quality control, a melting curve was generated between 60 °C and 99 °C. Gene expression data are presented relative to the housekeeping gene hypoxanthine-guanine phosphoribosyltransferase and relative to day 0 monolayer culture.

### Histological Stainings

Microtissues were gently flushed out from their microwells, concentrated, and fixed in 2% PFA overnight, embedded in 3% agarose, dehydrated, and embedded in paraffin overnight. Ectopic explants were fixed in 4% PFA for 4 hours, decalcified in ethylenediaminetetraacetic acid/PBS (pH 7.5) for 10 solution changes at 4 °C, dehydrated, and embedded in paraffin overnight, and sectioned at 5 µm thickness. Before histological staining, the slides were deparaffinized in Histoclear (Laborimpex).

For Safranin O/Fast green (Sigma) staining, sections were deparaffinized and dehydrated, counterstained with Hematoxylin (Merck, cat 6525) for 1 minute, briefly dipped in acid alcohol (1% HCL in 70% EtOH), rinsed in water, stained with 0.03% Fast Green (KLINIPATH, cat 80051) and then dipped in 1% glacial acetic acid followed by 7-minute staining in 0.25% SafraninO (KLINIPATH, cat 640780). Then the samples were washed in tap water, dehydrated with an ethanol series, cleared in histoclear, and mounted in Pertex for microscopy imaging. For Alcian blue staining, sections were deparaffinized, rehydrated, and stained with filtered Alcian blue solution for 30 minutes at room temperature. After washing, the slides were counterstained by Nuclear Fast Red for 5 minutes before washing, dehydrating, and mounting. Quantitative analysis and average intensity measurement were performed in Fiji (ImageJ) through the deconvolution plugin.

### Ethical Statement

All procedures on animal experiments were approved by the local ethical committee for Animal Research, KU Leuven. The animals were housed according to the regulations of the Animalium Leuven (KU Leuven).

### Statistical Analysis

All statistical analyses were performed using standard function in R (R core team). Statistical significance was defined at *P* < .05. Pairwise comparisons were done through a 2-sided, unpaired *t* test. Factor analysis was done by analysis of variance for the model: empty microwells=aspiration×dispension+time. Data are presented as mean and standard deviation from 4 samples. Symbols used are *P < .05, **P < .01, ***P < .001, and ****P < .0001.

## Results

### Automating Media Changes Requires Multifactorial Optimization

**Figure 1.**
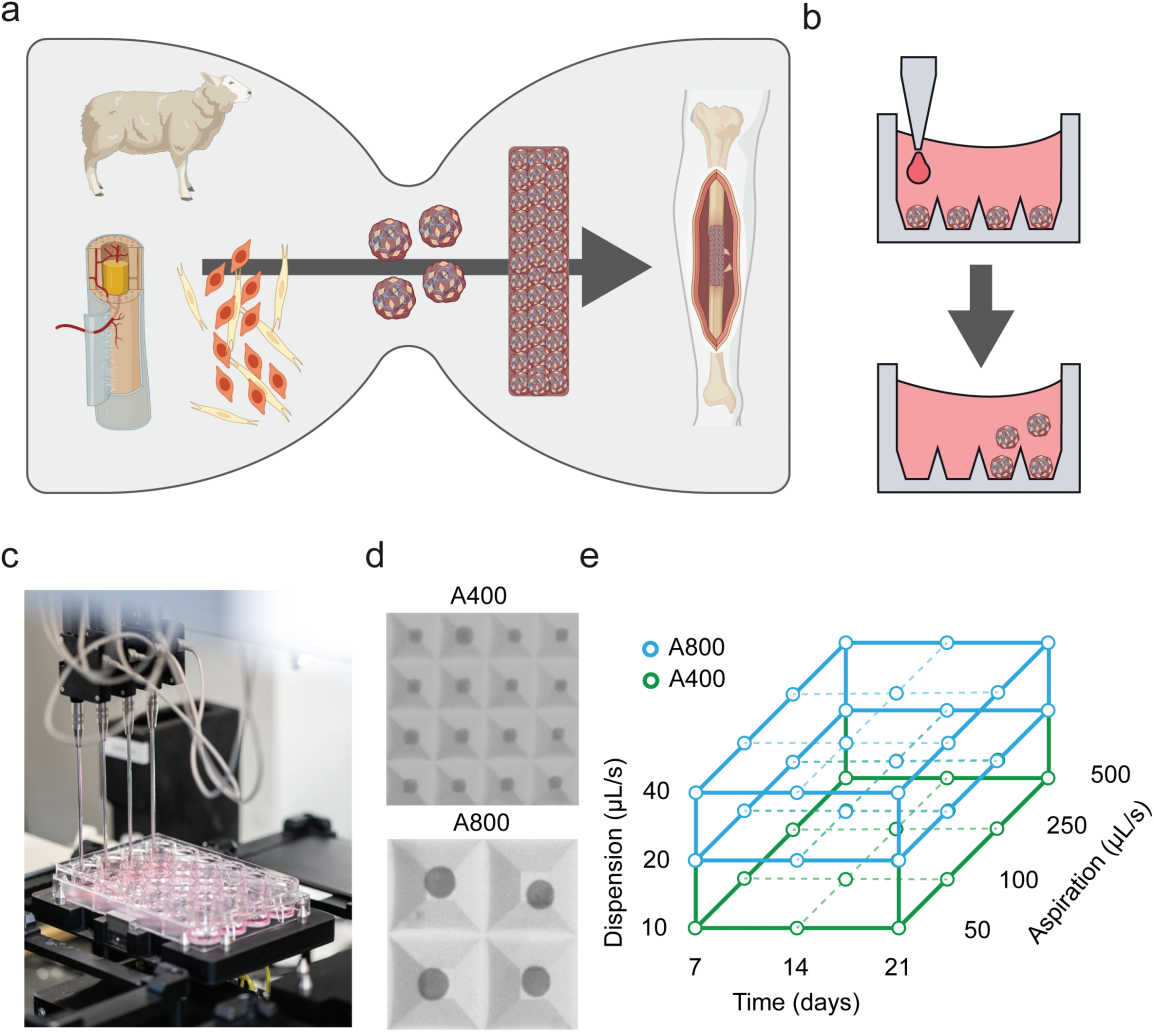
Experimental intention and experimental design. (**a**) A current bottleneck for microtissue-based long bone implants is a sufficient production of microtissues as building blocks. (**b**) Microtissue displacement as an effect of long-term culture. (**c**) Robotic media changes can be automated using a robotic liquid handling station. (**d**) Brightfield microscopy image showing a 1600 μm × 1600 μm field of view in microwell platforms A400 and A800. (**e**) Parameters of the experimental design. A400 = Aggrewell400 with 400 μm square microwells, A800 = Aggrewell800 with 800 μm square microwells. Created with BioRender.com.

Bottom-up tissue engineering requires the expansion of large cell quantities, followed by the production and long-term differentiation of homogeneous microtissues as building blocks. As shown in Fig. 1a, the scalability of microtissue production remains a bottleneck toward large implants. While static microwell platforms generate homogeneous microtissue populations, they require careful liquid handling to avoid microtissue displacement (Fig. 1b). We applied a liquid handling station, as shown in Fig. 1c, that is, part of an enclosed screening platform to explore the parameters that influence microtissue production speed, homogeneity, and yield. As shown in Fig. 1d, 2 alternative 24-well platforms were investigated: (1) aggrewell800 (A800; 300 microwells, 800 µm square, 1000 cells/microtissue) and aggrewell400 (A400; 1200 microwells, 400 µm square, 250 cells/microtissue), generating larger versus smaller microtissues. A full factorial experiment was designed as illustrated in Fig. 1e, and detailed in Fig. 3a, comparing 2 levels of media dispension speed, 4 levels of media aspiration speed for the 2 microwell platforms. Through noninvasive microscopic analysis, the effect of repeated media changes was monitored.

### Automated Image Analysis Pipeline

Microwell platforms enable the creation of homogeneous microtissues, yet their shape creates complex images through brightfield microscopy, hindering direct segmentation from the microwell platform. As a result, noninvasive monitoring over time remains challenging. We created a pipeline connecting machine learning-based image segmentation through pixel classification software (ilastik^66^) and object detection and data extraction software (cellprofiler^67^) to statistical data analysis scripts (R). These different modules are connected through jupyter notebooks written in python. All software is freely available and is gathered in one Docker container.

**Figure 2.**
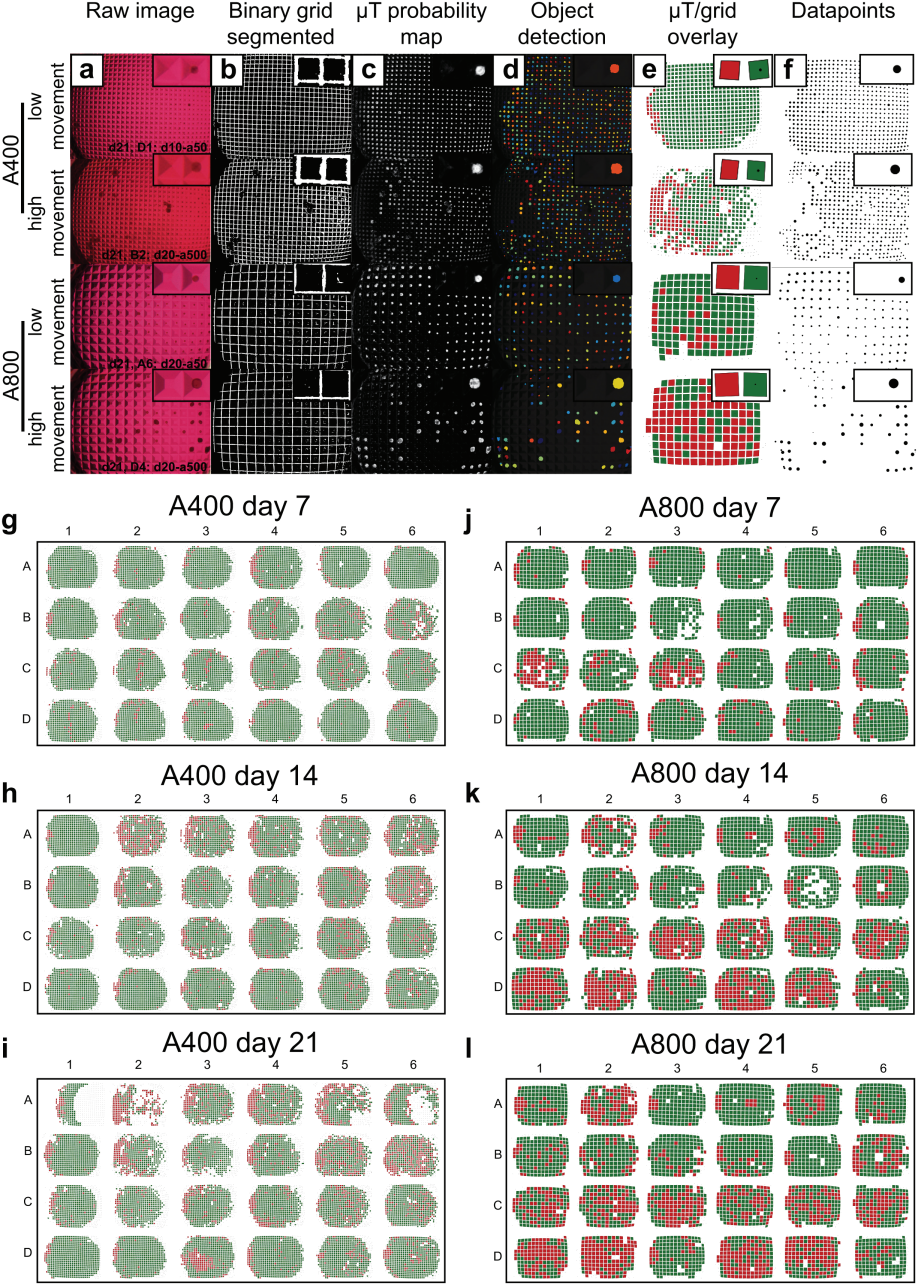
Image analysis pipeline. (**a**) Representative raw images of large and small microwells with high and low microtissue movement. (**b**) For each image, the microwell grid was segmented and (**c**) a microtissue probability map of all pixels was generated. (d) Individual spheroid objects were identified and (e) overlayed on the microwell grid. (**f**) Microtissue size distribution in a well. (**g-l**) 24 well plate digitalization for A400 and A800 and individual well tracking over time. Each microwell is colored according to whether it contains a microtissue or not.

The process, described in Fig. 2a--2f, starts with images containing any number of microtissues in square-shaped microwells. The images must be taken with identical settings to generate optimal results. In a first step, a pixel classification algorithm is trained in ilastik to predict whether a pixel belongs to a microtissue or background, resulting in a probability map. Then, a second pixel classification algorithm is trained to generate a mask of the microwell pattern. In cellprofiler, the microtissue probability map is thresholded, followed by object identification, thus segmenting individual microtissues. These microtissues are then described in relevant morphometric parameters including object shape (eg, location, area, diameter, and roundness) and location. Individual microwell contours are identified from the microwell pattern mask. For each microtissue, we calculated whether it is in a microwell or floating in-between. As a result, we could calculate which microwells are empty, and which contain more than one microtissue. Hence, microtissue displacements and empty microwells can be quantified and visualized per well (Fig. 2g--2l). The quality of the segmentation resulted in a dice coefficient of 0.885 for the training dataset, and 0.836 for the validation dataset. The amount of correctly identified empty microwells was 94% and 96%, respectively. As shown in Supplementary Fig. S1e, S1f, the number of empty microwells per well correlates to changes in roundness and size, making the percentage of empty microwells a fitting read-out to optimize liquid handling parameters. Moreover, as shown inSupplementary Fig. S1a--S1d, dotplots and density plots show size distributions within one image. Through a jupyter notebook, this pipeline can be used in batch mode to process hundreds of images containing thousands of microtissues. The data presented here contain information from 144 individual images and 51471 microtissues. Assuming a spherical shape in 3 dimensions, we can estimate microtissue volume from the 2D projection. With this approach, we performed particle analysis for the size distribution of individual microtissues over time as shown in Table 1. Projected volume calculations were used to investigate the effect of initial cell seeding density on the variability and final cartilage tissue volume produced. On average, we found that microtissues increased in size over time in both platforms. The variation in size, which is represented as the span measurement, indicates less size variability in A800 compared to A400. Furthermore, the total predicted tissue volume produced in one well is calculated as the sum of microtissue volumes, corrected for the percentage of microtissue displacement. We found that both platforms result in similar volume yields, here represented per 300 000 cells seeded per well.

**Table 1.** Summary statistics on microtissue size distribution and percentages of empty microwells.

Platform	Day	Microtissues identified	Microwells per well	Percentage empty microwells	Percentage filled microwells	Volume per microtissue (mm³)	D10 (mm³)	D50 (mm³)	D90 (mm³)	Span	Predicted tissue volume (mm³)
A400	7	14867	1200	8.82	91.18	0.0015	0.0009	0.0017	0.0033	1.4571	1.6014
	14	13210	1200	16.38	83.62	0.0017	0.0010	0.0020	0.0054	2.2674	1.7358
	21	12647	1200	21.11	78.89	0.0021	0.0012	0.0024	0.0070	2.4076	1.9432
A800	7	4014	300	10.62	89.38	0.0059	0.0033	0.0068	0.0160	1.8612	1.5851
	14	3574	300	26.80	73.20	0.0066	0.0036	0.0084	0.0162	1.5071	1.4487
	21	3158	300	29.23	70.77	0.0093	0.0048	0.0118	0.0318	2.2735	1.9731

Particle distribution parameters D10, D50, and D90 are derived from the ordered cumulative size distribution and indicate the microtissue size cutoff point where 10%, 50%, and 90% of microtissues respectively are below this size. The span, calculated as span=(D90−D10)/D50 is used to describe the distribution range. The predicted tissue volume per well is calculated as the amount of theoretical microwells per well times the average volume per microtissue, corrected for the amount of empty microwells.

### Automation and Optimization of Liquid Handling Parameters

Automated production of high volumes of microtissues is a major bottleneck in bottom-up tissue engineering (Fig. 1a). Automated medium changes could improve the scalability and homogeneity, yet one of the greatest challenges in long-term culture of microtissues in static well plates is their sensitivity to liquid handling. As illustrated in Fig. 1b, the microtissues can easily be displaced, leading to premature uncontrolled fusion into larger irregular tissues that complicate downstream processes. We used the automated liquid handling station as shown in Fig. 1c to maximize dispension and aspiration speeds for both small (A400) and large (A800) microwell platforms (Fig. 1d). In this way, the automated liquid handling station minimized microtissue displacement and time needed for media changes.

In the first design of experiment (DOE), we assessed the effect of aspiration speed, dispension speed, and needle depth in A400 through a 2^3^ full factorial design as shown inSupplementary Fig. S2a--S2e. In this experiment, we found no significant (*P* < .05) effects for the tested factor levels, but the data showed an indicative effect (*P* < .1) for both the dispension speed alone as the interaction of dispension speed with needle depth (Supplementary Fig. S2e). Both aspiration speed and needle depth were not significant in the range tested, but we noticed dripping for a 1 mm needle depth. Parameter testing requires a high amount of living microtissues, medium, and time. However, we found that gelatine beads, which are commonly used as a growth surface in suspension cultures, can be used as substitutes for living microtissues (Supplementary Fig. S2g). Because of the observed interaction between dispension speed and needle depth, we further optimized needle position by varying the needle depth and the distance from the edge of the well (Supplementary Fig. S2f). Because of the surface tension, the liquid has a concave shape. As a result, the needle can be placed further from the microtissues when placed more toward the side of the well. However, at 90% or more, the needle touches the well plate, disrupting the entire 24-well plate. An optimum was reached by placing the needle 2 mm below the liquid surface at 85% from the well center. As the needle followed the liquid level, a minimal number of spheroids were moved.

**Figure 3.**
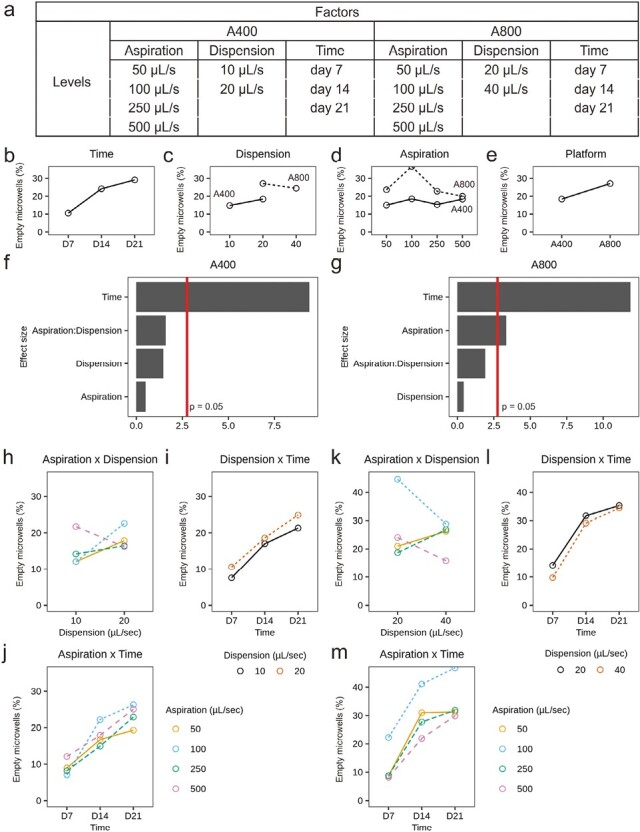
Design of Experiment (DE) setup and results. (**a**) Experimental design with the tested factors levels. (**b-e**) Main effects plots. (**f, g**) Statistical significance of main and interaction effects for A400 and A800 platforms. (**h-j**) Interaction plots for A400 and (**k-m**) interaction plots for A800.

A second DOE with 2 mixed factorial designs was set up as shown in Fig. 3a. As we suspected the larger microtissue platform (A800) to be less sensitive, we decreased the dispension speed of the smaller microtissue platform (A400), but kept the same for A800. Aspiration speed was not significant in the first DOE, so we increased the speed up to 500 µL/second, which is approximately 5× manual speed. On day 7, 14, and 21 brightfield images were taken to assess microtissue displacement and the cumulative effect over time. The automated image analysis pipeline was used for segmentation and data extraction as explained in Fig. 2a--2f. The main effects in Fig. 3b--3e show a significant average increase in empty microwells over time for both platforms. Dispension speed has no significant effect, but in A800, a significant effect was observed for aspiration speed. Especially for 100 µL/second, a high empty microwell percentage was measured. However, upon inspection of the distribution in the 24-well plate shown in Fig. 2j--2l, most wells with high levels of microtissue displacements locate in the bottom half of the well plate. As seen from the interaction effects in Fig. 3k, the 100 µL/second condition in the A800 platform is not in line with higher or lower aspiration speeds.

### Differentiation Potential of sPDCs

**Figure 4.**
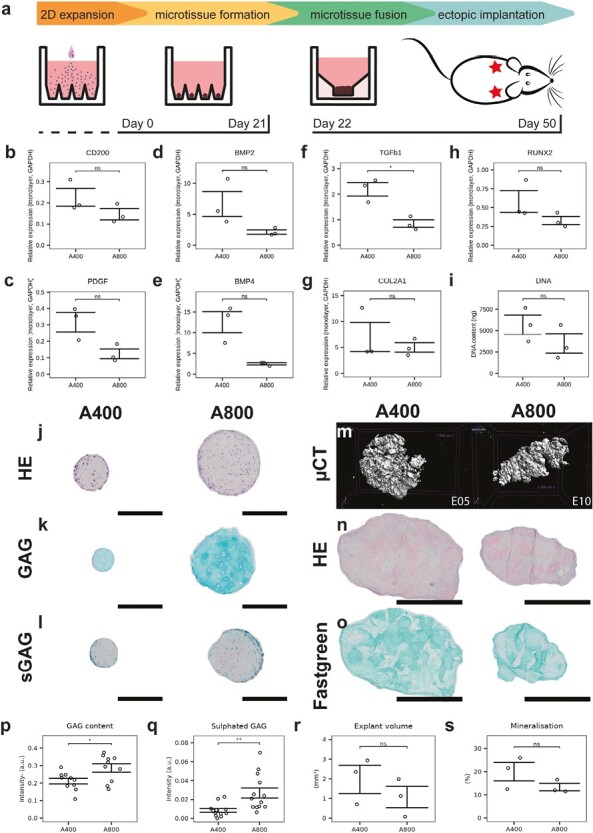
Biological characterization of microtissues and ectopic explants. (**a**) Protocol overview and timeline. (**b-g**) mRNA quantification of differentiation markers. (**i**) DNA quantification on day 21. Histological staining of microtissues on day 21 showing (**j**) hematoxylin-eosin, (**k**) Alcian blue, and (**l**) safranin O. Scale bars = 200 μm. (**m**) Mineralisation after 4 weeks ectopic implantation of microtissue constructs measured by microCT. Histological staining of explants showing (**n**) hematoxylin-eosin, and (**o**) safraninO-fastgreen staining of representative explants. Scale bars = 1 mm. (**p**) Glycosaminoglycan (GAG) quantification. (**q**) Sulphated glycosaminoglycan (sGAG) quantification. (**r**) Explant volumes. (**s**) Explant percentage mineralisation.

sPDCs were harvested and expanded in vitro for 8 passages. Supplementary Fig. S3a shows continued linear growth over 40 days. Supplementary Fig. S3b--S3e shows continued expression of periosteal progenitor markers, while osteoprogenitor markers RUNX2 and BMP2 show a slight decrease and connective tissue marker TGFβ shows a significant increase after passaging. To assess the differentiation potential of the sPDC, gene expression analysis and histological stainings were performed on day 21 microtissue populations (*n* = 3 wells). Fig. 4a recapitulates the experimental timeline. In Fig. 4b, 4c, we see a downregulation of proliferation and progenitor genes, CD200, and PDGF after 21 days compared to the same cells before differentiation. At the same time, Fig. 4d--4h shows that chondrogenic markers BMP2, BMP4, TGFb1, and COL2A1 were upregulated while osteogenic marker RUNX2 was downregulated in microtissue populations cultured in either platforms. Only TGFb1 showed a significant difference between the A400 and A800 platform. Also, as an identical amount of starting cells were seeded per well (300 000 cells/well), the DNA content shown in Fig. 4i after 21 days is the same for both platforms. We then analyzed the individual microtissues on a tissue level through immunohistochemistry (Fig. 4j--4l). Cells are homogeneously present throughout the microtissue with a thin layer on the outside. Alcian blue staining shows the secretion of cartilaginous glycosaminoglycans (GAG) in the extracellular matrix (Fig. 4k), but the intensity of GAG and sulphated GAGs, shown in Fig. 4l, was higher in larger microtissues. This was also confirmed with histological quantification as shown in Fig. 4p.

After 21 days, microtissues from 3 separate wells were collected, concentrated, and allowed to self-assemble for 24 hours, resulting in a construct that could be implanted ectopically for 4 weeks in nude mice to assess bone-forming potential. Micro-computed tomography (µCT) shows mineralization of all implants, but no cortical bone formation or bone marrow compartments (Fig. 4m). H&E staining shows remainders of microtissue shapes that are not yet remodeled (Fig. 4n). Yet, the explants stain positive for Fast Green (Fig. 4o), consistent with the mineralization seen in µCT. Quantification of explant size and mineralization in Fig. 4r, 4s show no significant differences between A400 and A800 implants, consistent with a similar implant volume as predicted in Table 1.

## Discussion

The use of microtissues and organoids as building blocks for the bottom-up engineering of larger tissues is rapidly increasing in tissue engineering.^71,72^ While this strategy shows promise, there is still a lack of automated technologies supporting robust culture and differentiation processes. These are hampered by complex manual protocols and limited noninvasive quality measurements. Therefore, to support translation of regenerative medicine, robust biomanufacturing processes are necessary.^73-75^ The development of such technologies will also enable the implementation of Quality by Design principles by linking measurable microtissue characteristics to the final product quality.^76^ The bottom-up TE approach inherently aspires to embed quality attributes that have been already present in the microtissue population within the final larger tissue products.

In this work, we explored the use of an academic high-content screening facility as a platform for robotics-based, long-time differentiation of ovine cartilaginous microtissues in nonadherent microwells. Here, we combined robotic media changes with automated brightfield-image–based noninvasive analysis. We applied this to run design of experiment (DOE) aiming at the optimization of liquid handling parameters to enable long differentiation processes. It has previously been shown that automating methods using robotics is an efficient way to reduce human error and operator-dependent variability in laboratory operations.^77^ One of the challenges for culturing microtissues in nonadherent well plates is that over time they are prone to move, leading to uncontrolled fusion and the development of large agglomerates.^25^ This can result in tissue heterogeneity and influence the synchronization of differentiation cascades over time leading also to the development of nondifferentiated tissue locations.^19^ By introducing robotic medium changes, we investigated the effect of needle positioning, dispension, and aspiration speed in ranges close to and beyond manual speed and found no statistically significant effect. Therefore, robotic media changes can be time-saving. Still, a continued mapping of the design space in relation to the critical product profile will include also other factors such as media change frequency and volumes. We did detect a significant time dependence, which we attribute to the manual handling of the microwell plates between incubator, microscope, and media change station. Further integrating robotic plate handling and imaging is thus considered to be the next step. To date the adoption of robotics for cell therapy and regenerative medicine applications has been carried out for the expansion of single-cell populations such as adult MSC expanded in bioreactor systems.^42^ Moreover, robotics-based expansion and differentiation of induced pluripotent stem cells toward retinal pigment epithelial cells showed a high level of cell purity and functionality.^78,79^ In this work, we take a step further as we explore a bioprocess problem related to nonadherent microtissue protocols, as opposed to protocols encompassing adherent cell and organoid types, which has been largely unexplored.

The definition of biological critical quality attributes by morphometrics, spectroscopic techniques, or secreted proteins could be the next step to ensure a predictive outcome upon implantation.^80-85^ On line, noninvasive monitoring techniques, such as the current approach, can produce large quantities of data on the process and biology that can be used as input for machine learning algorithms. Automated imaging technologies have been implemented to characterize 2D cell culture processes such as expansion of MSCs^86,87^ but also for the automated imaging of spheroid and microtissue structures.^15-18,25,88^ The use of automated image analysis can contribute to further minimizing human error and variability which can also greatly improve the manufacturing process as it feeds back to the process parameters used during manufacturing.^89^ In this work, the automated brightfield image segmentation and characterisation of microtissues were achieved with supervised opensource software linked through jupyter notebooks and a docker environment for reproducibility and ease of use. For example, users can train a classifier to identify patterns in the segmented microtissue images for predictive maintenance.^90^ A machine learning classifier can predict the failure of the microtissue manufacturing process and support informed decision-making in the early stages of cell culture. Ultimately, this can help process design and enhance process control and ultimately contribute to process optimization.^91-94^ As these microtissue populations serve as active raw materials for living implants, morphological properties are important considerations for the appropriate selection of downstream bio-assembly methods.^95,96^

Finally, for implants targeting the healing of long-bone fractures, the ovine model is frequently used and would be the animal of choice to validate TE products before the transition to a clinical trial.^97,98^ Hence, we used ovine periosteal cells to assess their differentiation potential and bone-forming capacity. We see encouraging differentiation and the formation of cartilaginous microtissues that mineralize upon implantation showing regions of bone with blood vessel invasion. Compared to human periosteal cell microtissues,^19^ the ovine periosteal microtissues display lower tissue maturation kinetics. Ectopic implantation shows the onset of bone formation, yet we observe a slower cartilage-to-bone transition, with a lack of bone marrow compartments and a large portion of mineralizing cartilage.^72,99^ Further investigation of these implants at orthotopic sites, such as critical size segmental tibial defects, will be required in future studies.

In conclusion, a stepwise automation of manual tasks, and eventual fully automated biomanufacturing of cartilaginous microtissue populations is becoming increasingly feasible, enabling next steps toward the translation process. Here, we show how robotic media changes combined with multifactorial DOE, noninvasive imaging, and automated image analysis can be used to optimize large-scale biomanufacturing of microtissue building blocks, while being compatible with noninvasive quality monitoring.

## Supplementary Material

szad091_suppl_Supplementary_Figures_S1-S3

## Data Availability

The data that support the findings of this study are available upon reasonable request from the authors.

## Software and Code Availability

All software is defined in a docker container which is freely available at https://hub.docker.com/r/gnasello/spheroid-env and all notebooks used to generate the image processing workflow are available in Github (https://github.com/isaakdecoene/spheroid_classifier).

## References

[1] Zanoni M , Piccinini F, Arienti C, et al. 3D tumor spheroid models for in vitro therapeutic screening: a systematic approach to enhance the biological relevance of data obtained. Sci Rep. 2016;6(1):1-11. http://sourceforge.net/p/revisp/26752500 10.1038/srep19103 PMC4707510

[2] Kim S , Kim EM, Yamamoto M, Park H, Shin H. Engineering multi-cellular spheroids for tissue engineering and regenerative medicine. Adv Healthc Mater. 2020;9(23):1-18. 10.1002/adhm.202000608. 32734719

[3] Ragelle H , Naba A, Larson BL, et al. Comprehensive proteomic characterization of stem cell-derived extracellular matrices. Biomaterials. 2017 ;128:147-159. 10.1016/j.biomaterials.2017.03.008 28327460 PMC8191742

[4] Liu D , Chen S, Win Naing M. A review of manufacturing capabilities of cell spheroid generation technologies and future development [Internet]. Biotechnol Bioeng. 2021;118:542-554. https://onlinelibrary.wiley.com/doi/full/10.1002/bit.27620 33146407 10.1002/bit.27620

[5] Takebe T , Wells JM. Organoids by design. Science. 2019;364:956-959. https://www.science.org/doi/10.1126/science.aaw7567 31171692 10.1126/science.aaw7567 PMC8212787

[6] Hofer M , Lutolf MP. Engineering organoids. Nat Rev Mater. 2021;6(5):402-420. 10.1038/s41578-021-00279-y 33623712 PMC7893133

[7] Burdis R , Chariyev-Prinz F, Browe DC, et al. Spatial patterning of phenotypically distinct microtissues to engineer osteochondral grafts for biological joint resurfacing. Biomaterials. 2022; 289; 121750. https://linkinghub.elsevier.com/retrieve/pii/S0142961222003908 10.1016/j.biomaterials.2022.121750 36084483

[8] Lipsitz YY , Timmins NE, Zandstra PW. Quality cell therapy manufacturing by design. Nat Biotechnol. 2016;34(4):393-400. 10.1038/nbt.3525 27054995

[9] Petry F , Salzig D. Large-scale production of size-adjusted beta-cell spheroids in a fully controlled stirred-tank reactor. Processes. 2022;10(5):861. https://www.mdpi.com/2227-9717/10/5/861/htm

[10] Lim D , Renteria ES, Sime DS, et al. Bioreactor design and validation for manufacturing strategies in tissue engineering. Biodes Manuf. 2021;5(1):43-63. 10.1007/s42242-021-00154-3 35223131 PMC8870603

[11] Caprio N , Burdick JA. Engineered biomaterials to guide spheroid formation, function, and fabrication into 3D tissue constructs. Acta Biomater. 2022;165:4-18. 10.1016/j.actbio.2022.09.052 36167240 PMC10928646

[12] Van Loo B , Ten Den SA, Araújo-Gomes N, et al. Mass production of lumenogenic human embryoid bodies and functional cardiospheres using in-air-generated microcapsules. Nat Commun. 2023;14(1):1-15. https://www.nature.com/articles/s41467-023-42297-0 37865642 10.1038/s41467-023-42297-0 PMC10590445

[13] Freeman FE , Pitacco P, van Dommelen LHA, et al. 3D bioprinting spatiotemporally defined patterns of growth factors to tightly control tissue regeneration. Sci Adv. 2020;6(33):1-16. 10.1126/sciadv.abb5093 PMC7428335 32851179

[14] Rossen NS , Anandakumaran PN, zur Nieden R, et al. Injectable therapeutic organoids using sacrificial hydrogels. iScience 2020;23(5):101052. 10.1016/j.isci.2020.101052 32353766 PMC7191221

[15] Lacalle D , Castro-Abril HA, Randelovic T, et al. SpheroidJ: an open-source set of tools for spheroid segmentation. Comput Methods Programs Biomed. 2021;200(1):105837. 10.1016/j.cmpb.2020.105837 33221056

[16] Grexa I , Diosdi A, Harmati M, et al. SpheroidPicker for automated 3D cell culture manipulation using deep learning. Sci Rep. 2021;11(1):1-11. https://www.nature.com/articles/s41598-021-94217-1 34285291 10.1038/s41598-021-94217-1 PMC8292460

[17] Deckers T , Hall GN, Papantoniou I, Aerts JM, Bloemen V. A platform for automated and label-free monitoring of morphological features and kinetics of spheroid fusion. Front Bioeng Biotechnol. 2022;10:1441. https://www.frontiersin.org/articles/10.3389/fbioe.2022.946992/full 10.3389/fbioe.2022.946992 PMC9461702 36091464

[18] Shirai K , Kato H, Imai Y, et al. The importance of scoring recognition fitness in spheroid morphological analysis for robust label-free quality evaluation. Regen Ther. 2020;14(6):205-214. 10.1016/j.reth.2020.02.004 32435672 PMC7229423

[19] Nilsson Hall G , Rutten I, Lammertyn J, et al. Cartilaginous spheroid-assembly design considerations for endochondral ossification: towards robotic-driven biomanufacturing. Biofabrication 2021;13(4):045025. 10.1088/1758-5090/ac2208 34450613

[20] Schindelin J , Arganda-Carreras I, Frise E, et al. Fiji: an open-source platform for biological-image analysis. Nat Methods. 2012;9(7):676-682. 10.1038/nmeth.2019 22743772 PMC3855844

[21] Sofroniew N , Lambert T, Evans K, Nunez-Iglesias J, Bokota G, Winston P, et al. napari: a multi-dimensional image viewer for Python. 2022. 10.5281/zenodo.8115575

[22] Wieland F , Schumacher A, Roumans N, van Blitterswijk C, Lapointe V, Rademakers T, et al. Methodological approaches in aggregate formation and microscopic analysis to assess pseudoislet morphology and cellular interactions [version 2; peer review: 2 approved]. 2022. 10.12688/openreseurope.14894.1 PMC10446072 37645341

[23] Decoene I , Herpelinck T, Geris L, Luyten FP, Papantoniou I. Engineering bone-forming callus organoid implants in a xenogeneic-free differentiation medium. Front Chem Eng. 2022;4(892190):71.

[24] Eintracht J , Harding P, Lima Cunha D, Moosajee M, Ratnayaka JA. Efficient embryoid-based method to improve generation of optic vesicles from human induced pluripotent stem cells. 2022. 10.12688/f1000research.108829.1 PMC9218590 35811797

[25] Brandenberg N , Hoehnel S, Kuttler F, et al. High-throughput automated organoid culture via stem-cell aggregation in microcavity arrays. Nat Biomed Eng. 2020;4(9):863-874. 10.1038/s41551-020-0565-2 32514094

[26] Lenas P , Moos MJ, Luyten FP. Developmental engineering: a new paradigm for the design and manufacturing of cell-based products part I: from three-dimensional cell growth to biomimetics of in vivo development. Tissue Eng Part B Rev. 2009;15(4):381-394. 10.1089/ten.TEB.2008.0575 19505199

[27] Lenas P , Luyten FP. An emerging paradigm in tissue engineering: from chemical engineering to developmental engineering for bioartificial tissue formation through a series of unit operations that simulate the in vivo successive developmental stages. Ind Eng Chem Res. 2011;50(2):482-522. 10.1021/ie100314b

[28] Sheehy EJ , Mesallati T, Kelly L, et al. Tissue engineering whole bones through endochondral ossification: regenerating the distal phalanx. Biores Open Access. 2015;4(1):229-241. 10.1089/biores.2015.0014 26309799 PMC4540120

[29] Mendes LF , Tam WL, Chai YC, et al. Combinatorial analysis of growth factors reveals the contribution of bone morphogenetic proteins to chondrogenic differentiation of human periosteal cells. Tissue Eng Part C Methods 2016;22(5):473-486. 10.1089/ten.TEC.2015.0436 27018617

[30] Nilsson Hall G , Mendes LF, Gklava C, et al. Developmentally engineered callus organoid bioassemblies exhibit predictive in vivo long bone healing. Adv Sci. 2019;7(2):1-16. 10.1002/advs.201902295 PMC6974953 31993293

[31] Martini L , Fini M, Giavaresi G, Giardino R. Sheep model in orthopedic research: a literature review. Comp Med. 2001;51(4):292-299. 11924786

[32] Rousseau CF , Maciulaitis R, Sladowski D, Narayanan G. Cell and gene therapies: European view on challenges in translation and how to address them. Front Med (Lausanne). 2018;5:158. 10.3389/fmed.2018.00158 29911104 PMC5992383

[33] ten Ham RMT , Hoekman J, Hövels AM, et al. Challenges in advanced therapy medicinal product development: a survey among companies in Europe. Mol Ther Methods Clin Dev. 2018;11(12):121-130. 10.1016/j.omtm.2018.10.003 30456217 PMC6234262

[34] Silva DN , Chrobok M, Ahlén G, et al. ATMP development and pre-GMP environment in academia: a safety net for early cell and gene therapy development and manufacturing. Immunooncol Technol. 2022;16(100099):1-7. 10.1016/j.iotech.2022.100099 PMC9650027 36389443

[35] Wilson AJ , Brown N, Rand E, Genever PG. Attitudes towards standardization of mesenchymal stromal cells—a qualitative exploration of expert views. Stem Cells Transl Med 2023;12(11):745-757. 10.1093/stcltm/szad056 37713249 PMC10630078

[36] Sun W , Starly B, Daly AC, et al. The bioprinting roadmap. Biofabrication. 2020;12(2):022002. 10.1088/1758-5090/ab5158 32031083

[37] Groschl M , Markus A, Leyers S, et al. A liquid handling robot for robust and reproducible preparation of standard and quality control samples in bioanalysis. Adv Robot Autom. 2017;06(01):1-3.

[38] Dettinger P , Kull T, Arekatla G, et al. Open-source personal pipetting robots with live-cell incubation and microscopy compatibility. Nat Commun. 2022;13(1):1-12. https://www.nature.com/articles/s41467-022-30643-7 35637179 10.1038/s41467-022-30643-7 PMC9151679

[39] Mehesz AN , Brown J, Hajdu Z, et al. Scalable robotic biofabrication of tissue spheroids. Biofabrication. 2011;3(2):025002. 10.1088/1758-5082/3/2/025002 21562365 PMC4699548

[40] Hussain W , Moens N, Veraitch FS, et al. Reproducible culture and differentiation of mouse embryonic stem cells using an automated microwell platform. Biochem Eng J. 2013;77(100):246-257. 10.1016/j.bej.2013.05.008 23956681 PMC3741632

[41] Ochs J , Biermann F, Piotrowski T, et al. Fully automated cultivation of adipose-derived stem cells in the StemCellDiscovery—a robotic laboratory for small-scale, high-throughput cell production including deep learning-based confluence estimation. Processes. 2021;9(4):575. https://www.mdpi.com/2227-9717/9/4/575/htm

[42] Ochs J , Hanga MP, Shaw G, et al. Needle to needle robot-assisted manufacture of cell therapy products. Bioeng Transl Med. 2022; 7(3):e10387. https://onlinelibrary.wiley.com/doi/full/10.1002/btm2.10387 36176619 10.1002/btm2.10387 PMC9472012

[43] Doulgkeroglou MN , Di Nubila A, Niessing B, et al. Automation, monitoring, and standardization of cell product manufacturing. Front Bioeng Biotechnol. 2020;8(7):811. 10.3389/fbioe.2020.00811 32766229 PMC7381146

[44] Tristan CA , Ormanoglu P, Slamecka J, et al. Robotic high-throughput biomanufacturing and functional differentiation of human pluripotent stem cells. Stem Cell Rep. 2021;16(12):3076-3092. 10.1016/j.stemcr.2021.11.004 PMC8693769 34861164

[45] Boussaad I , Cruciani G, Bolognin S, et al. Integrated, automated maintenance, expansion and differentiation of 2D and 3D patient-derived cellular models for high throughput drug screening. Sci Rep. 2021;11(1):1439. 10.1038/s41598-021-81129-3 33446877 PMC7809482

[46] Kanda GN , Tsuzuki T, Terada M, et al. Robotic search for optimal cell culture in regenerative medicine. Elife. 2022;11:e77007. 10.7554/eLife.77007 35762203 PMC9239686

[47] Ohta A , Kawai S, Pretemer Y, et al. Automated cell culture system for the production of cell aggregates with growth plate-like structure from induced pluripotent stem cells. SLAS Technol. 2023;28(6):433-441. http://slas-technology.org/article/S2472630323000523/fulltext 37562511 10.1016/j.slast.2023.08.002

[48] Krieger J , Nießing B, König N, et al. Implementation of an automated manufacturing platform for engineering of functional osteochondral implants. Procedia CIRP. 2022;110:32-35. 10.1016/j.procir.2022.06.008

[49] Novelli G , Spitalieri P, Murdocca M, Centanini E, Sangiuolo F. Organoid factory: the recent role of the human induced pluripotent stem cells (hiPSCs) in precision medicine. Front Cell Dev Biol. 2023;10:1059579. 10.3389/fcell.2022.1059579 36699015 PMC9869172

[50] Aguilar IN , Smith LJ, Olivos DJ, et al. Scaffold-free bioprinting of mesenchymal stem cells with the regenova printer: optimization of printing parameters. Bioprinting. 2019;15):e00048. 31457110 10.1016/j.bprint.2019.e00048 PMC6711201

[51] Shudo Y , MacArthur JW, Kunitomi Y, et al. Three-dimensional multilayered microstructure using needle array bioprinting system. Tissue Eng Part A. 2020;26(5-6):350-357. 10.1089/ten.TEA.2019.0313 32085692 PMC7476375

[52] Dalton PD , Woodfield TBF, Mironov V, Groll J. Advances in hybrid fabrication toward hierarchical tissue constructs [Internet]. Adv Sci. 2020;7:1902953. Cited June 16, 2021. www.advancedscience.com 10.1002/advs.201902953 PMC7284200 32537395

[53] Kikuchi T , Kino-oka M, Wada M, et al. A novel, flexible and automated manufacturing facility for cell-based health care products: tissue factory. Regen Ther. 2018;9:89-99. 10.1016/j.reth.2018.08.004 30525079 PMC6223031

[54] Smiatek J , Jung A, Bluhmki E. Towards a digital bioprocess replica: computational approaches in biopharmaceutical development and manufacturing. Trends Biotechnol. 2020;38(10):1141-1153. 10.1016/j.tibtech.2020.05.008 32518043

[55] Lee E , Shah D, Porteus M, Wright JF, Bacchetta R. Design of experiments as a decision tool for cell therapy manufacturing. Cytotherapy. 2022;24(6):590-596. 10.1016/j.jcyt.2022.01.009 35227602

[56] Toms D , Deardon R, Ungrin M. Climbing the mountain: experimental design for the efficient optimization of stem cell bioprocessing. J Biol Eng. 2017 ;11(1):35. 10.1186/s13036-017-0078-z 29213303 PMC5712411

[57] Kuterbekov M , Machillot P, Baillet F, et al. Design of experiments to assess the effect of culture parameters on the osteogenic differentiation of human adipose stromal cells. Stem Cell Res Ther. 2019;10(1):256. 10.1186/s13287-019-1333-7 31412950 PMC6694725

[58] Ladner YD , Armiento AR, Kubosch EJ, Snedeker JG, Stoddart MJ. Optimization of loading protocols for tissue engineering experiments. Sci Rep. 2022;12(1):5094. 10.1038/s41598-022-08849-y 35332169 PMC8948220

[59] Yasui R , Sekine K, Taniguchi H. Clever experimental designs: shortcuts for better ipsc differentiation. Cells. 2021;10(12):3540. 10.3390/cells10123540 34944048 PMC8700474

[60] Bharadwaz A , Dhar S, Jayasuriya AC. Full factorial design of experiment-based and response surface methodology approach for evaluating variation in uniaxial compressive mechanical properties, and biocompatibility of photocurable PEGDMA-based scaffolds. Biomed Mater. 2023;18(2):025019. 10.1088/1748-605X/acb7bd 36720161

[61] Dellaquila A , Campodoni E, Tampieri A, Sandri M. Overcoming the design challenge in 3D biomimetic hybrid scaffolds for bone and osteochondral regeneration by factorial design. Front Bioeng Biotechnol. 2020;8:743. 10.3389/fbioe.2020.00743 32775321 PMC7381347

[62] Pisani S , Genta I, Modena T, et al. A proof of concept to define the parameters affecting poly-l-lactide-*co*-poly-ε-caprolactone shape memory electrospun nanofibers for biomedical applications. Drug Deliv Transl Res. 2023;13(2):593-607. 10.1007/s13346-022-01218-2 35978259 PMC9794533

[63] Kuchemüller KB , Pörtner R, Möller J. Design of cell expansion processes for adherent-growing cells with mDOE-workflow. Eng Life Sci. 2023;23(5):e2200059. 10.1002/elsc.202200059 37153028 PMC10158623

[64] Saucourt C , Vogt S, Merlin A, et al. Design and validation of an automated process for the expansion of peripheral blood-derived CD34+ cells for clinical use after myocardial infarction. Stem Cells Transl Med. 2019;8(8):822-832. 10.1002/sctm.17-0277 31037857 PMC6646685

[65] Rivera-Ordaz A , Peli V, Manzini P, Barilani M, Lazzari L. Critical analysis of cGMP large-scale expansion process in bioreactors of human induced pluripotent stem cells in the framework of quality by design. Biodrugs. 2021;35(6):693-714. 10.1007/s40259-021-00503-9 34727354 PMC8561684

[66] Berg S , Kutra D, Kroeger T, et al. ilastik: interactive machine learning for (bio)image analysis. Nat Methods. 2019;16(12):1226-1232. 10.1038/s41592-019-0582-9 31570887

[67] Lamprecht MR , Sabatini DM, Carpenter AE. CellProfilerTM: free, versatile software for automated biological image analysis. Biotechniques. 2007;42(1):71-75. 10.2144/000112257 17269487

[68] Stirling DR , Swain-Bowden MJ, Lucas AM, et al. CellProfiler 4: improvements in speed, utility and usability. BMC Bioinf. 2021;22(1):1-11. https://bmcbioinformatics.biomedcentral.com/articles/10.1186/s12859-021-04344-9 10.1186/s12859-021-04344-9 PMC8431850 34507520

[69] Bradski G. The OpenCV Library. Dr Dobb’s J Softw Tools. 2000;120:122-125.

[70] Edzer JP , Roger SB. Classes and methods for spatial data in R. R News. 2005;5(2):9-13.

[71] Geris L , Papantoniou I. The third era of tissue engineering: reversing the innovation drivers. Tissue Eng Part A. 2019;25(11-12):821-826. 10.1089/ten.TEA.2019.0064 30860432

[72] Burdis R , Kelly DJ. Biofabrication and bioprinting using cellular aggregates, microtissues and organoids for the engineering of musculoskeletal tissues. Acta Biomater. 2021;126:1-14. 10.1016/j.actbio.2021.03.016 33711529

[73] Lambrechts T , Papantoniou I, Rice B, et al. Large-scale progenitor cell expansion for multiple donors in a monitored hollow fibre bioreactor. Cytotherapy. 2016;18(9):1219-1233. 10.1016/j.jcyt.2016.05.013 27421744

[74] Moutsatsou P , Ochs J, Schmitt RH, Hewitt CJ, Hanga MP. Automation in cell and gene therapy manufacturing: from past to future. Biotechnol Lett. 2019;41(11):1245-1253. 10.1007/s10529-019-02732-z 31541330 PMC6811377

[75] Rafiq QA , Twomey K, Kulik M, et al. Developing an automated robotic factory for novel stem cell therapy production. Regen Med. 2016;11(4):351-354. 10.2217/rme-2016-0040 27168080

[76] McCorry MC , Reardon KF, Black M, et al. Sensor technologies for quality control in engineered tissue manufacturing. Biofabrication. 2022;15(1):012001. 10.1088/1758-5090/ac94a1 PMC10283157 36150372

[77] Archibald PRT , Chandra A, Thomas D, et al. Comparability of automated human induced pluripotent stem cell culture: a pilot study. Bioprocess Biosyst Eng. 2016;39(12):1847-1858. 10.1007/s00449-016-1659-9 27503483 PMC5050253

[78] Regent F , Morizur L, Lesueur L, et al. Automation of human pluripotent stem cell differentiation toward retinal pigment epithelial cells for large-scale productions. Sci Rep. 2019;9(1):1-11. https://www.nature.com/articles/s41598-019-47123-6 31337830 10.1038/s41598-019-47123-6 PMC6650487

[79] Kanda GN , Tsuzuki T, Terada M, et al. Robotic search for optimal cell culture in regenerative medicine. Elife. 2022;11(e77007):11. 10.7554/eLife.77007. PMC9239686 35762203

[80] Baradez MO , Biziato D, Hassan E, Marshall D. Application of Raman spectroscopy and univariate modelling as a process analytical technology for cell therapy bioprocessing. Front Med (Lausanne). 2018;5:14. 29556497 10.3389/fmed.2018.00047 PMC5844923

[81] Mercier SM , Rouel PM, Lebrun P. Process analytical technology tools for perfusion cell culture. 2016;25-35.

[82] Caldwell J , Wang W, Zandstra PW. Proportional-integral-derivative (PID) control of secreted factors for blood stem cell culture. PLoS One. 2015;10(9):e0137392. 10.1371/journal.pone.0137392 26348930 PMC4562642

[83] Chilmonczyk MA , Kottke PA, Stevens HY, Guldberg RE, Fedorov AG. Dynamic mass spectrometry probe for electrospray ionization mass spectrometry monitoring of bioreactors for therapeutic cell manufacturing. Biotechnol Bioeng. 2019;116(1):121-131. 10.1002/bit.26832 30199089 PMC6310154

[84] Marklein RA , Lam J, Guvendiren M, Sung KE, Bauer SR. Functionally-relevant morphological profiling: a tool to assess cellular heterogeneity. Trends Biotechnol. 2018;36(1):105-118. 10.1016/j.tibtech.2017.10.007 29126572

[85] Suyama T , Takemoto Y, Miyauchi H, et al. Morphology-based noninvasive early prediction of serial-passage potency enhances the selection of clone-derived high-potency cell bank from mesenchymal stem cells. Inflamm Regen. 2022;42(1):1-13. https://inflammregen.biomedcentral.com/articles/10.1186/s41232-022-00214-w 36182958 10.1186/s41232-022-00214-w PMC9526913

[86] Imai Y , Iida M, Kanie K, Katsuno M, Kato R. Label-free morphological sub-population cytometry for sensitive phenotypic screening of heterogenous neural disease model cells. Sci Rep. 2022;12(1):1-13. https://www.nature.com/articles/s41598-022-12250-0 35710681 10.1038/s41598-022-12250-0 PMC9203459

[87] Matsuoka F , Takeuchi I, Agata H, et al. Morphology-based prediction of osteogenic differentiation potential of human mesenchymal stem cells. PLoS One. 2013;8(2):e55082. 10.1371/journal.pone.0055082 23437049 PMC3578868

[88] Deckers T , Lambrechts T, Viazzi S, et al. High-throughput image-based monitoring of cell aggregation and microspheroid formation. PLoS One. 2018;13(6):e0199092. https://journals.plos.org/plosone/article?id=10.1371/journal.pone.0199092 29953450 10.1371/journal.pone.0199092 PMC6023212

[89] Allenby MC , Woodruff MA. Image analyses for engineering advanced tissue biomanufacturing processes. Biomaterials. 2022;284(May):121514. 10.1016/j.biomaterials.2022.121514 35413510

[90] Wuest T , Weimer D, Irgens C, Thoben KD. Machine learning in manufacturing: advantages, challenges, and applications. 2016;4(1):23-45. https://www.tandfonline.com/doi/abs/10.1080/21693277.2016.1192517

[91] Odeh-Couvertier VY , Dwarshuis NJ, Colonna MB, et al. Predicting T-cell quality during manufacturing through an artificial intelligence-based integrative multiomics analytical platform. Bioeng Transl Med. 2022;7(2):e10282. 10.1002/btm2.10282 35600660 PMC9115702

[92] Srinivasan M , Thangaraj SR, Ramasubramanian K, Thangaraj PP, Ramasubramanian KV. Exploring the current trends of artificial intelligence in stem cell therapy: a systematic review. Cureus. 2021;13(12):e20083. https://pubmed.ncbi.nlm.nih.gov/34873560/34873560 10.7759/cureus.20083 PMC8635466

[93] Hort S , Herbst L, Bäckel N, et al. Toward rapid, widely available autologous CAR-T cell therapy – artificial intelligence and automation enabling the smart manufacturing hospital. Front Med (Lausanne). 2022;9(June):1605. 10.3389/fmed.2022.913287. PMC9207622 35733863

[94] Watson BM , Kasper K, Mikos -AG, et al. The future of bone regeneration: integrating AI into tissue engineering. Biomed Phys Eng Express. 2021;7(5):052002. 10.1088/2057-1976/ac154f. 34271556

[95] Banerjee D , Singh YP, Datta P, et al. Strategies for 3D bioprinting of spheroids: a comprehensive review. Biomaterials. 2022;291:121881. 10.1016/j.biomaterials.2022.121881 36335718

[96] Daly AC , Davidson MD, Burdick JA. 3D bioprinting of high cell-density heterogeneous tissue models through spheroid fusion within self-healing hydrogels. Nat Commun. 2021;12(1):1-13. https://www.nature.com/articles/s41467-021-21029-2 33531489 10.1038/s41467-021-21029-2 PMC7854667

[97] Lammens J , Maréchal M, Geris L, et al. Warning about the use of critical-size defects for the translational study of bone repair: analysis of a sheep tibial model. Tissue Eng Part C Methods 2017;23(11):694-699. 10.1089/ten.TEC.2017.0147 28594312

[98] Sparks DS , Saifzadeh S, Savi FM, et al. A preclinical large-animal model for the assessment of critical-size load-bearing bone defect reconstruction. Nat Protoc. 2020;15(3):877-924. 10.1038/s41596-019-0271-2 32060491

[99] Grigoryan A , Zacharaki D, Balhuizen A, et al. Engineering human mini-bones for the standardized modeling of healthy hematopoiesis, leukemia, and solid tumor metastasis. Sci Transl Med. 2022;14(666):1-16. https://www.science.org/doi/10.1126/scitranslmed.abm6391 10.1126/scitranslmed.abm6391 36223446

