# Supplementary material for: Robotics-Driven Manufacturing of Cartilaginous Microtissues for Skeletal Tissue Engineering Applications
Source: Stem Cells Transl Med. 2024 Jan 13;13(3):278–92. doi: 10.1093/stcltm/szad091 (PMC10940839; doi:10.1093/stcltm/szad091)
Supplement: szad091_suppl_Supplementary_Figures_S1-S3 [file szad091_suppl_supplementary_figures_s1-s3.pdf]

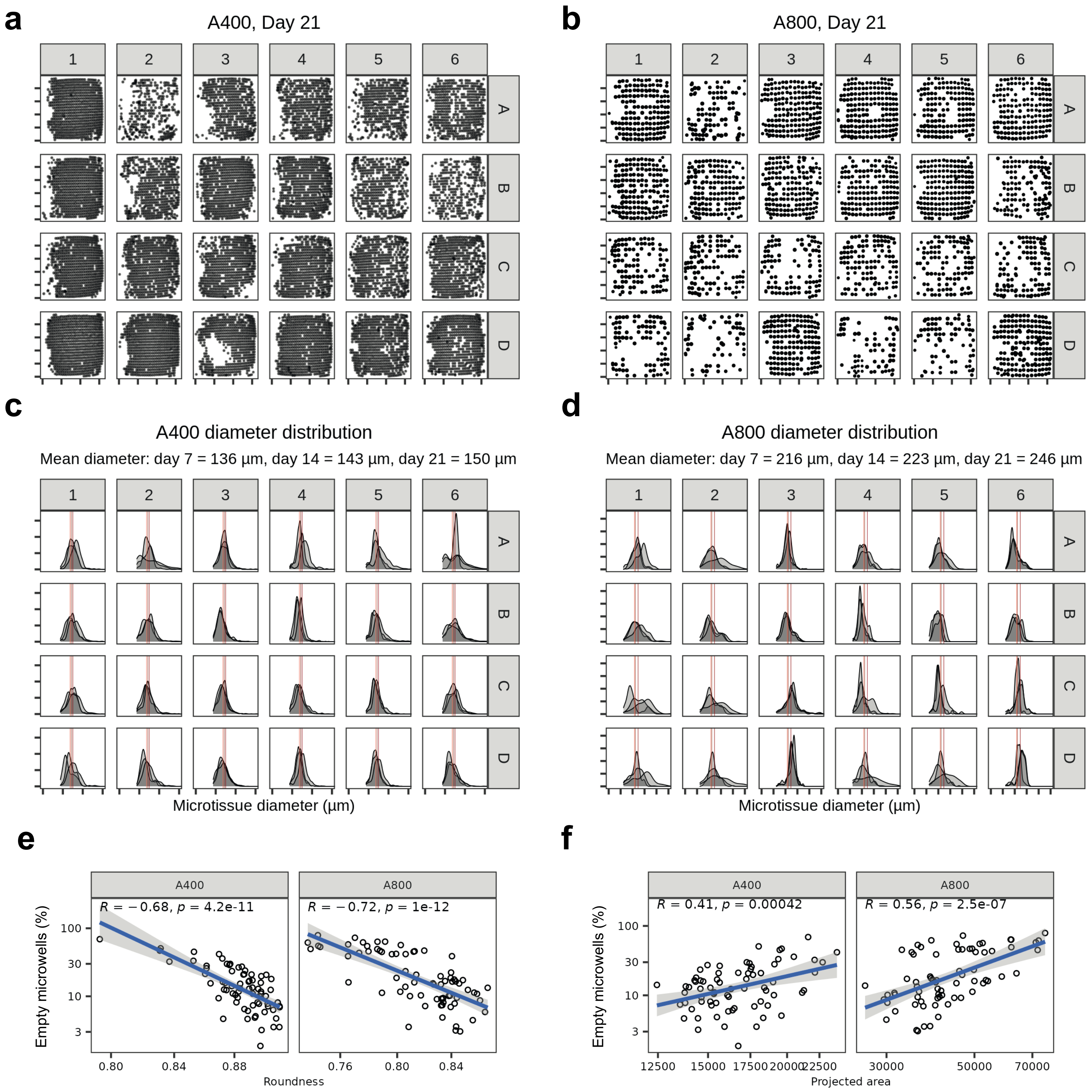

Supplemental figure 1. Microtissues as datapoints for process monitoring. (a-b) microtissue locations and diameter (µm). (c-d) Density plots showing the diameter of microtissues in each well in the 24-well pate over time. (e) Correlation between average amount of microwells in a well and average microtissue roundness. (f) Correlation between average amount of microwells in a well and the average size of microtissues in a well.

a

First DoE: Aggrewell400

Factor	Min value	Max value
Aspiration speed	20 µL/sec	50 µL/sec
Dispension speed	10 µL/sec	40 µL/sec
Needle depth	1 mm	2 mm

b

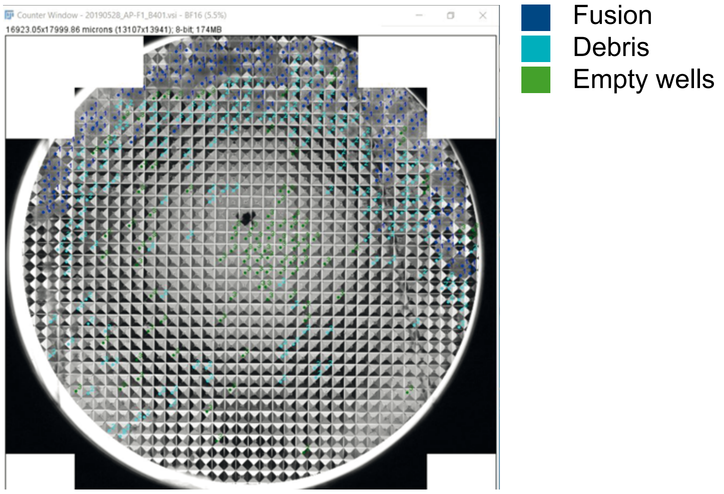

c

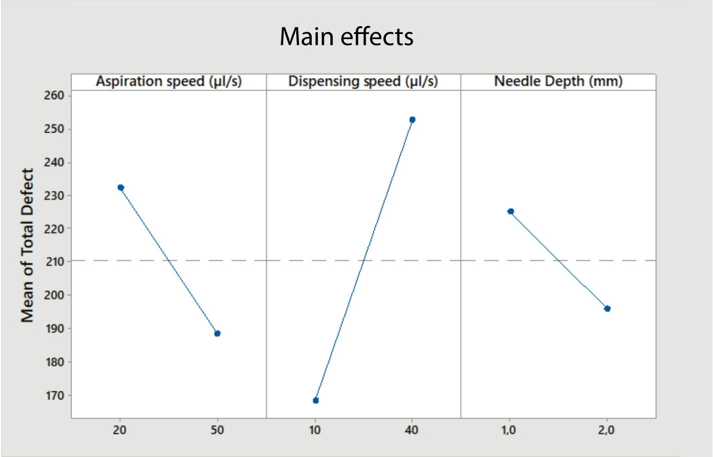

d

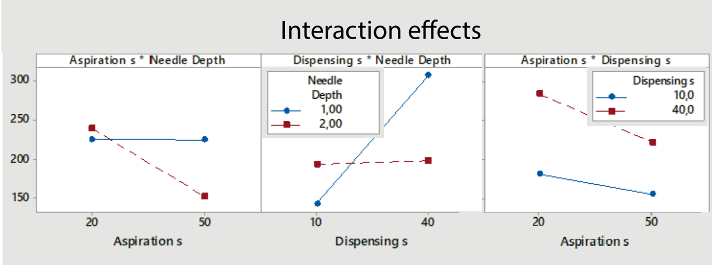

e

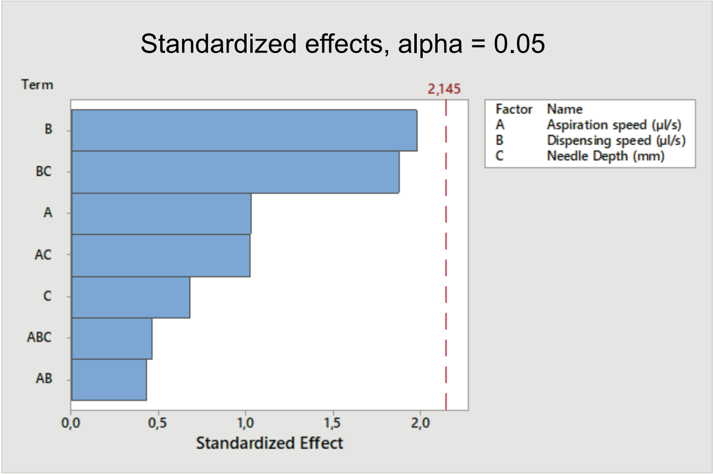

f

Optimisation of needle placement

Needle placement (% from right side)	needle depth from surface (mm)	drops	spheroid movement	plate movement
50	-3	0	+++'	0
50	-2,5	0	+++'	0
50	-2,4	0	++'	0
50	-2,3	+	++'	0
80	-2,4	0	++'	0
85	-2	0	+'	0
90	-2	0	+'	+'
95	-2	0	+++'	+++'

g

Parameter testing with gelatin beads

Before pipetting After pipetting

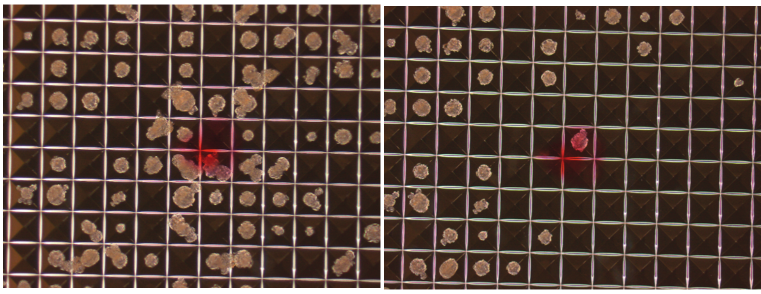

Supplemental figure 2. First parameter screening and optimisation. (a) DoE design showing factors and levels. (b) Example of manual analysis strategy. (c-e) DoE analysis results showing (c) main effects, (d) interaction effects, and (e) statistical significance. (f) summary table of empirical testing of needle placement parameters. (g) brightfield images of gelatine beads used during optimisation of needle placement with microwell size is 400 µm.

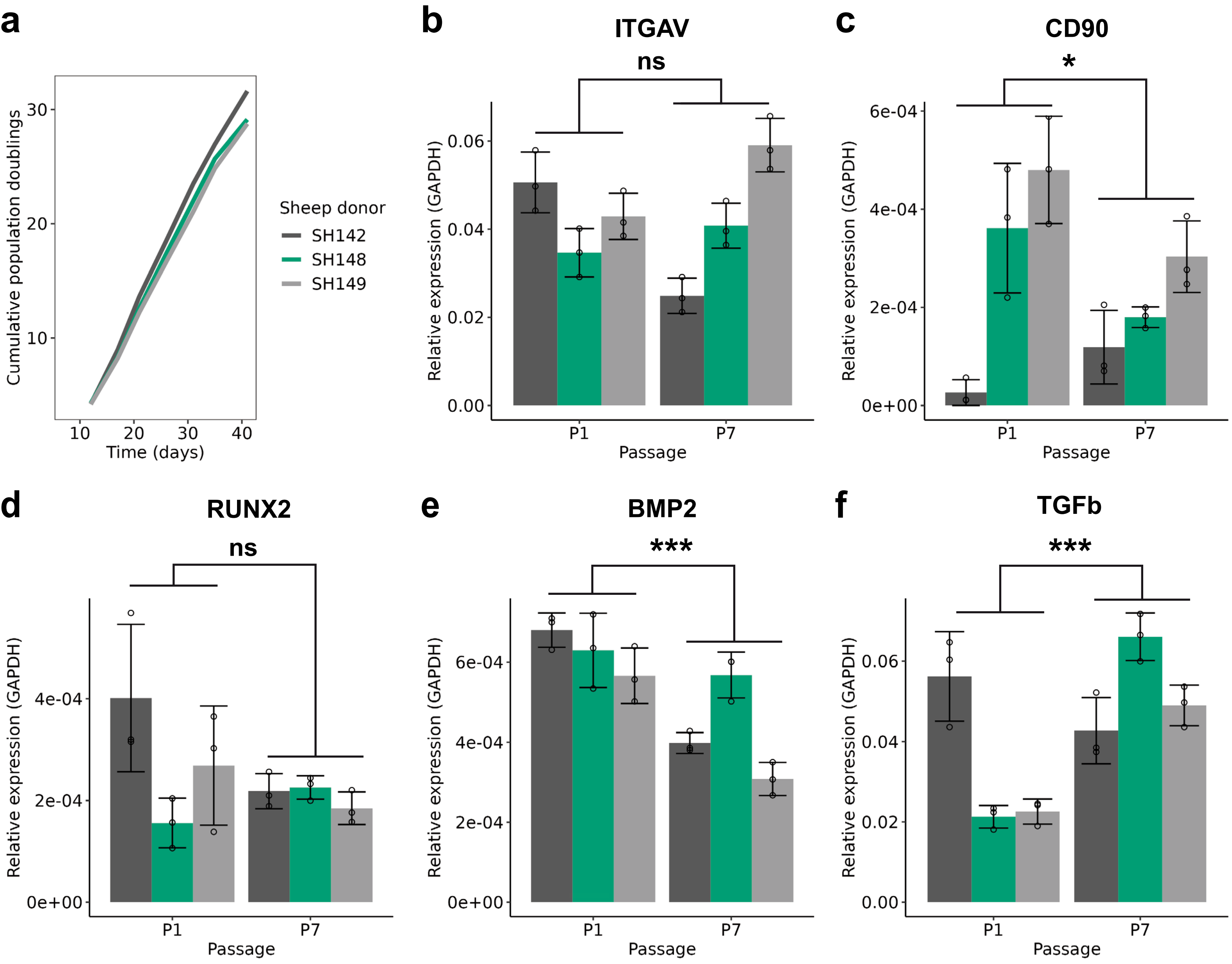

Supplemental figure 3. Characterisation of sheep periosteum-derived cells during expansion. (a) growth curves of periosteum cell from three sheep donors. (b-c) Gene expression of periosteal progenitor markers at passage 1 versus 7. (d-e) Gene expression of osteoprogenitor markers. (f) Gene expression of connective tissue marker. p =*0,05, **0,01, ***0,001
